# Enhanced Starch-Hydrocolloid Synergism Through Critical Melting and Freeze-Thawing: Mechanism of Structural Weakening and Chain Reassociation

**DOI:** 10.3390/foods15142523

**Published:** 2026-07-16

**Authors:** Chen Zhang, Sheng-Yi Wang, Zirui Xu, Chu-Yun Wu, Yi-Tong Zhang, Yong-Li Wang, Yu-Jie Wang, Jian-Ya Qian

**Affiliations:** School of Food Science and Engineering, Yangzhou University, Huayang Xilu 196, Yangzhou 225127, China; mz120252313@stu.yzu.edu.cn (S.-Y.W.); 253601209@stu.yzu.edu.cn (Z.X.); mx120241324@stu.yzu.edu.cn (C.-Y.W.); mz120251350@stu.yzu.edu.cn (Y.-T.Z.); 243603218@stu.yzu.edu.cn (Y.-L.W.); 243601323@stu.yzu.edu.cn (Y.-J.W.); jyqian@yzu.edu.cn (J.-Y.Q.)

**Keywords:** starch, hydrocolloids, critical melting, structure, gel property

## Abstract

The study aimed to enhance the interchain entanglement between tapioca starch (TS) and hydrocolloids by partially weakening starch structure and promoting chain reassociation, using critical melting combined with freeze-thawing treatment (CMFT). Compared to simple blends (SBL), CMFT induced partial structural disruption and facilitated soluble starch release, likely promoting chain entanglement with hydrocolloids to form large reorganized clusters with a rough granular surface. The observed structural and functional changes support this interpretation. CMFT reduced relative crystallinity from 25.88% (TS) to ~20%, while preserving granular integrity, and increased gelatinization temperatures by ~3 °C. The CMFT-prepared composite showed significantly improved pasting properties, with PV and FV rising from 2526.50 and 2087.00 (TS) to ~2700 and ~2400 mPa·s, respectively. CMFT transformed the weak, elongated TS paste into a cohesive, structurally integrated network with ~3-fold higher gel hardness and substantially reduced digestibility (RS content increased from 43.3% to approximately 60%). The study provides an effective strategy to enhance TS-hydrocolloid interaction by partially weakening starch structure and chain reassociation for designing starch-based ingredients with tailored functional properties.

## 1. Introduction

Hydrocolloids are commonly used functional ingredients in starch-based food matrices to engineer desired textural attributes, enhance stability, and control moisture mobility of food products [1,2,3,4]. It is a green way to modify starch structure and is considered an effective strategy for developing clean-label products. Physical blending of starch with hydrocolloids is a widely used approach in the food industry. However, the functional outcomes of simple blends are often variable and difficult to predict, even with fixed combinations of starch and hydrocolloid types [5,6]. This can lead to contradictory effects on critical functional properties, such as paste viscosity, gelatinization behavior, and gel strength. In a simple mixture system, hydrocolloids predominantly interact with starch on the surface of starch granules via reversible hydrogen bonding and steric effects, which are less stable and more prone to induce phase separation in a mixture system [7,8].

In simple blend systems, interactions between starch and hydrocolloids are primarily limited on the surface of starch granules by an intermolecular steric association or surface weak hydrogen bonding [9]. These interfacial interactions are inherently weak and can be readily disrupted by competitive binding of water molecules, often leading to phase separation in the mixture and limiting their interactions. To overcome these limitations, a fundamental transition from surface adsorption to molecular entanglement is required. Many studies have also reported that the intra- and inter-chain interactions between starch and hydrocolloids are more stable and effective than the intermolecular steric interaction [10,11]. In other words, effective interaction requires more available molecular segments or short chains. Despite this mechanistic understanding, practical physical strategies that can promote such interchain integration while preserving granular integrity remain limited. For example, annealing primarily enhances crystalline order without generating substantial soluble chains for entanglement, whereas ultrasonic treatment can release soluble chains but often causes extensive granular fragmentation, which compromises the functional performance of starch in applications such as thickening or texture modification. Chemical approaches, while effective, are less desirable in the context of clean-label product development. It was therefore hypothesized that if a physical strategy could partially weaken the starch structure to release soluble chains while maintaining granular integrity, it might substantially enhance starch–hydrocolloid interactions.

Critical melting and freeze-thawing (CMFT) treatment was recently reported to be an effective method to partially disrupt the starch granular structure to release soluble chains for starch structure modification [11,12,13]. Critical melting (CM) is defined as heating the starch suspension to the onset gelatinization temperature (To) of native starch, at which the initial disruption of crystalline regions begins while granular integrity is largely preserved and effectively promotes soluble starch release. This targeted thermal energy partially disrupts the granular semi-crystalline structure, thereby promoting soluble starches release without causing granular disintegration. Subsequently, the freeze-thawing (FT) facilitates entanglement through ice crystal mechanical forces and ice-templating, further promoting the reassociation of the released starch chains into a cohesive, three-dimensional network.

Native tapioca starch (TS) is a widely available bioresource, characterized by a well-defined thermal transition and high swelling power, which makes it more susceptible to thermal treatment and easily releases soluble starches during processing [14]. Moreover, TS typically produces pastes with low and unstable viscosity, as well as weak and structurally unstable gels, which limits its widespread use in food systems, such as structured sauces, gelled desserts, and starch-based snacks. Carboxymethyl cellulose (CMC) and xanthan gum (XG) are two commonly used hydrocolloids with distinct structural features. CMC is a linear anionic polysaccharide, while XG is a branched heteropolysaccharide that adopts a helical conformation in solution. Both are widely employed in food systems for their thickening, stabilizing, and water-binding properties [15,16,17]. In particular, the linear structure of CMC may facilitate entanglement with amylose chains, whereas the helical and charged nature of XG could promote associative interactions with starch components.

Therefore, in the present study, tapioca starch (TS) was combined with xanthan gum (XG) or carboxymethyl cellulose (CMC) and was subjected to critical melting combined with freeze–thawing (CMFT) to explore the potential for enhancing their interaction. Critical melting aimed to partially disrupt and weaken the starch structure and release soluble chains to interact with XG or CMC, while freeze–thawing aimed to further promote their reassociation and facilitate interchain entanglement. The effects of CMFT in promoting TS-hydrocolloids interactions were systematically investigated by analyzing granular morphology, physicochemical properties, multi-scale structure, paste and gel properties, and the digestibility of the composites. The objective was to elucidate the structural modifications and interaction mechanisms induced by CMFT in TS-hydrocolloid systems, thereby providing a scientific foundation for tailoring the functionality of starch-based foods.

## 2. Materials and Methods

### 2.1. Materials

Native tapioca starch (TS) was purchased from Litian Industry Co., Ltd. (Chengdu, China). The moisture, ash, crude protein, amylose, and amylopectin contents were 11.82%, 0.15%, 0.5%, 18.5%, and 81%, respectively. Carboxymethyl cellulose (CMC) and xanthan gum (XG) were purchased from Macklin Biochemical Co., Ltd. (Shanghai, China), and Yuanye Biotechnology Co., Ltd. (Shanghai, China), respectively. The molecular weight and apparent viscosity of CMC were 7000 kDa and 2500–4500 mPa·s (1%, 25 °C, on a dry basis), while for XG, the corresponding values were 1.68 × 10^6^ Da and 1200–1600 mPa·s, respectively.

### 2.2. Sample Preparation

CMC and XG were separately dissolved in deionized water at room temperature with continuous magnetic stirring (500 rpm) for 30 min to ensure complete hydration before mixing with starch. The hydrocolloid solutions were then added to starch powder to achieve final concentrations of 0.1%, 0.3%, and 0.5% (*w*/*w*, based on starch dry weight). The starch-to-water ratio was maintained at 2:3 (*w*/*w*). The suspensions were stirred continuously for 30 min at room temperature. This suspension served as the common starting material for SBL, FT, and CMFT, the only difference among these three groups was the application of subsequent thermal and/or freeze–thaw treatments. Then, the mixture was heated at the onset gelatinization temperature (To) of TS in a water bath for 1 h with magnetic stirring.

The onset gelatinization temperature (To) of native TS was determined by differential scanning calorimetry (DSC 8500, PerkinElmer, Waltham, MA, USA) according to the method described in Section 2.6 (To: 65.52 ± 1.58 °C). Critical melting was performed by heating the starch-hydrocolloid suspension in a temperature-controlled water bath (Julabo, Seelbach, Germany) maintained at To ± 0.5 °C. The heating process was conducted with continuous magnetic stirring at 300 rpm for 1 h. Temperature was monitored using a calibrated thermocouple immersed in the sample to ensure temperature stability. After heating, the samples were frozen at −20 °C for 12 h and subsequently thawed in a water bath at 25 °C for 2 h.

After their respective treatments, all three treated groups (SBL, FT, and CMFT) were rapidly dried in a convection oven at 60 °C for 8 h to ensure uniform moisture content, a condition selected to minimize starch retrogradation based on preliminary experiments, and the dried cakes were ground into fine powders, and passed through a 100-mesh sieve. For clarity and comparison, in this work, a simple blend of starch and hydrocolloid without treatment was designated as “SBL”, the mixture subjected only to freeze–thaw was designated as “FT”, and the mixture subjected to critical melting combined with freeze–thawing was designated as “CMFT”.

### 2.3. Soluble Starch Release

The release of soluble starch was determined using an iodine-binding method described by Raza et al. [18] with minor modifications. After treatment according to Section 2.2, the mixture was cooled to room temperature and then centrifuged for 20 min at 10,000× *g*, and collect the supernatant. A fixed volume of the supernatant was diluted with deionized water to a defined volume, then mixed with an equal volume of iodine solution (0.2% I_2_ in 2% KI) and incubated for 10 min. The absorbance was measured at 650 nm using a UV-Vis spectrophotometer (INESA Analytical Instrument Co., Ltd., Shanghai, China).

### 2.4. Granular and Physicochemical Properties

#### 2.4.1. Morphology and Birefringence (LM)

Granule morphology and birefringence of the composite were observed under a polarizing light microscope (BA3, 10Pol, Motic Industrial Group Co., Ltd., Xiamen, China). Morphology and birefringence of samples were heated in a water bath at 50, 70, and 90 °C for 30 min, respectively and observed. For each specimen, five randomly selected fields were documented at 40× magnification.

#### 2.4.2. Scanning Electron Microscopy (SEM)

Surface microstructure was characterized using a using field-emission scanning electron microscope (GeminiSEM 300, Carl Zeiss, Jena, Germany) at an acceleration voltage of 5.0 kV. Powdered specimens were affixed to aluminum stubs and coated with a thin gold layer under vacuum using a BAL-TEC SCD 500 coater (BAL-TEC AG, Balzers, Liechtenstein). Representative images were captured at a magnification of 3000×.

#### 2.4.3. Water Solubility Index (WSI) and Swelling Power (SP)

Each sample powder was suspended in deionized water at 10 g/kg (dry basis) and heated in a water bath at 70 °C for 30 min, respectively. After centrifugation at 1610× *g* for 30 min, the supernatant was collected an dried at 105 °C to constant weight, while the precipitated pellet was weighed immediately. The following parameters were then calculated according to Equations (1) and (2), respectively:

(1) $$\mathrm{WSI}\;(\%)=\frac{W_{1}}{W}\times 100$$

(2) $$\mathrm{SP}\;(g/g)=\frac{W_{2}\times 100}{W(100-WSI)}$$

where W (g) is the initial weight of the dry sample (on dry basis), W_1_ (g) is the weight of the dried supernatant, and W_2_ (g) is the weight of the wet precipitate.

#### 2.4.4. Paste Transmittance

Paste transmittance was evaluated following the procedure of Zhang et al. [19] with minor modifications. Aqueous dispersions (10 g/kg, *w*/*w*) were heated in a boiling water bath for 15 min, then stored at 25 °C. Transmittance at 620 nm was recorded after 0, 24, and 48 h of storage using a UV-Vis spectrophotometer (INESA Analytical Instrument Co., Ltd., Shanghai, China), with deionized water as the blank.

### 2.5. Structural Properties

#### 2.5.1. X-Ray Diffractometry (XRD)

X-ray diffractograms were recorded on a D8 Advance diffractometer (Bruker AXS, Karlsruhe, Germany) using the protocol described by Zhang et al. [13] with modifications. Samples were packed into an aluminum pan and then scanned from 3° to 40° (2θ) at 4°/min, with the generator operating at 40 kV and 30 mA. Relative crystallinity (RC%) was calculated from the diffractograms using Jade 6.0 software, according to Equation (3):

(3) $$\mathrm{RC}\;\left(\%\right)=\left(\frac{\mathrm{IC}}{IC+IA}\right)\times 100\%$$

where IC and IA are the cumulative diffraction intensity of the crystalline and amorphous region, respectively.

#### 2.5.2. Small-Angle X-Ray Scatterometer (SAXS)

Lamellar structure were measured using a NanoSTAR small-angle X-ray scattering system (Bruker AXS, Karlsruhe, Germany) with Cu Kα radiation (λ = 0.154 nm) generated at 50 kV and 50 mA. Scattering data were acquired over a q-range of 0.2–1.4 nm^−1^, where q = 4π sin θ/λ. The lamellar repeat distance (dBragg) was derived from the scattering peak position using Woolf Bragg’s equation: d = 2π/q [20]. Peak areas (Ap) were computed with Origin 7.5.

#### 2.5.3. Fourier Transform Infrared Spectroscopy (FTIR)

Short-range ordered structure of the composite was analyzed using a Fourier transform infrared spectrometer (Cary 610/670, Varian, Salt Lake City, UT, USA) according to the method reported in Ye et al. [21] with minor modifications. The samples were ground with spectroscopic-grade KBr and compressed into transparent pellets under vacuum. The pellets were scanned 64 times over the range of 400–4000 cm^−1^ at a resolution of 4 cm^−1^. Background spectra were collected with a pure KBr pellet and subtracted automatically. The spectra obtained were analyzed using OMNIC professional software (Version 8.3, Thermo Nicolet Corp., Madison, WI, USA) after removing the background interference. The absorbance intensity ratios at 1047/1022 cm^−1^ (R_1_) and 1022/995 cm^−1^ (R_2_) were calculated from the deconvoluted spectra.

### 2.6. Differential Scanning Calorimetry (DSC)

Thermal properties were determined using a DSC 8500 calorimeter (PerkinElmer, MA, USA). 5 mg of samples (dry basis) were mixed with 10 μL of deionized water in sealed aluminum pans and equilibrated at 4 °C overnight. Heating from 25 to 100 °C was performed at 5 °C/min, and thermograms were analyzed for onset (To), peak (Tp), and conclusion (Tc) temperatures, and enthalpy (ΔH) were determined from the thermograms.

### 2.7. Paste and Gel Properties

#### 2.7.1. Rapid Visco-Analysis (RVA)

Pasting properties were measured using a Rapid Visco-Analyzer (Newport Scientific Pty, Warriewood, NSW, Australia). Sample suspensions (70 g/kg dry solids, 30 g total weight) were subjected to the following profile: holding at 50 °C for 1 min (960 rpm), heating to 95 °C at 13 °C/min with a 3 min hold, then cooling to 50 °C at 13 °C/min with a 4 min hold. Parameters recorded included peak viscosity (PV), trough (TV), breakdown (BD), setback (SB), final viscosity (FV), and pasting temperature and time (PT^1^ and PT^2^).

#### 2.7.2. Paste Length Properties

Paste length was determined using the method of Zhang et al. [22] with modifications. The powders were suspended in deionized water (80 g/kg, dry solids, *w*/*w*), then pasted in a boiling water bath with continuous magnetic stirring for 10 min. The prepared hot starch paste was transferred into a Petri dish and allowed to cool to room temperature. A spoonful of the paste was lifted to a uniform height, then tilted at angles of 0°, 45°, and 90° to observe the paste behavior and evaluate the cohesiveness of its internal network. Furthermore, a light-colored gloved hand was used to vertically press down on the starch paste for 5 s, and then slowly lifted upwards until the paste broke. The length of the starch paste was recorded by placing a ruler next to the paste vertically. The length of the starch paste, from the point of initial contact to the point where it broke, was recorded using a digital camera.

#### 2.7.3. Gel Morphology

For gel observation, pastes were prepared as described in Section 2.7.2 and poured into plastic molds (15 mm diameter × 10 mm depth). After standing at room temperature for 24 h, gel morphology was photographed using a digital camera (EOS 50D, Canon Inc., Tokyo, Japan). The overall shape and surface morphology of the prepared gel were photographed using a digital single-lens reflex camera (EOS 50D, Canon Inc., Tokyo, Japan).

#### 2.7.4. Surface Adhesion Properties of the Gel

Gel adhesiveness was qualitatively evaluated by rolling each gel specimen over a 10 cm distance on a bench surface using a gloved hand. The glove was then sprayed with iodine solution to reveal any starch residue transferred from the gel.

#### 2.7.5. Texture Properties of Gel

Texture properties were measured using a texture analyzer (TA. TOUCH, BosinTech, Shanghai, China), fitted with a 25 mm cylindrical probe. Two-cycle compression was applied at 1.0 mm/s to 40% deformation. Each sample was tested at least six times.

#### 2.7.6. Microstructure of Gel

Gel microstructure was observed using an emission scanning electron microscope (GeminiSEM 300, Carl Zeiss, Jena, Germany) at 5.0 kV. Gels were freeze-dried, thinly sectioned, mounted onto stubs, and sputter-coated with gold under vacuum (BAL-TEC SCD 500, Liechtenstein) The micrographs were captured at a magnification of 1000× magnification.

### 2.8. In Vitro Starch Digestibility

Starch digestibility was following the method of Englyst et al. [23] and Lv et al. [5] with slight modifications. 200 mg of the sample suspended in 15 mL of sodium acetate buffer (0.1 M, pH 5.2) and equilibrated at 37 °C for 10 min in a shaking water bath. Digestion was started by adding 10 mL of a freshly prepared enzyme mixture containing pancreatin (porcine, 8 × USP, 5 g/L, Sigma P1750, Sigma-Aldrich, St. Louis, MO, USA) and amyloglucosidase (*Aspergillus niger*, 300 U/mL, Sigma A7095, Sigma-Aldrich) in the same buffer; enzyme activity was verified against a starch standard prior to use. The mixture was incubated at 37 °C with shaking. Aliquots (0.5 mL) withdrawn at 0, 20, and 120 min were mixed with 4 mL of ethanol to stop the reaction and centrifuged at 3000× *g* for 10 min. Glucose content in the supernatant was measured using a glucose oxidase-peroxidase assay kit (GOPOD, Megazyme, Bray, Ireland) at 505 nm. The contents of rapidly digestible starch (RDS%), slowly digestible starch (SDS%), and resistant starch (RS%) were calculated according to Equations (4)–(6), respectively:

(4) $$RDS\left(\%\right)=\left[\frac{\left(G20-G0\right)\times 0.9}{TS}\right]\times 100$$

(5) $$SDS\left(\%\right)=\left[\frac{\left(G120-G20\right)\times 0.9}{TS}\right]\times 100$$

(6) $$RS\left(\%\right)=\left[\frac{TS-RDS-SDS}{TS}\right]\times 100$$

where TS is the total starch content, G0 is the free glucose content in the starch suspension before enzymolysis (mg), and G20 and G120 are the glucose contents after 20 and 120 min of hydrolysis (mg), respectively. The factor 0.9 converts glucose to starch equivalents. All measurements were performed in triplicate.

### 2.9. Statistical Analysis

Each technological treatments measurement was performed in triplicate. Data are presented as means ± standard deviations. The data obtained were subjected to analysis of variance using Duncan’s multiple range test (*p* < 0.05) performed with SPSS 25 (SPSS Institute Inc., Cary, NC, USA). Figures were prepared with Origin 8.05 (Stat-Ease Inc., Minneapolis, MN, USA).

## 3. Results and Discussion

### 3.1. Soluble Starch Release, Granular Morphology, Birefringence, and Surface Microstructure of Composite

**Figure 1:**
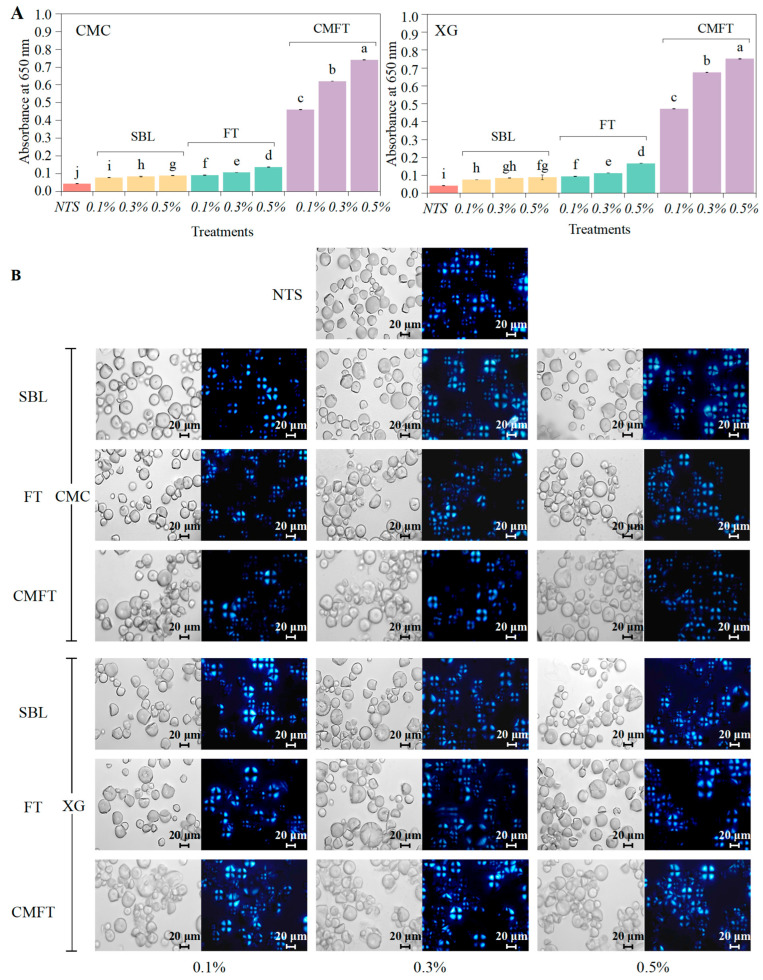

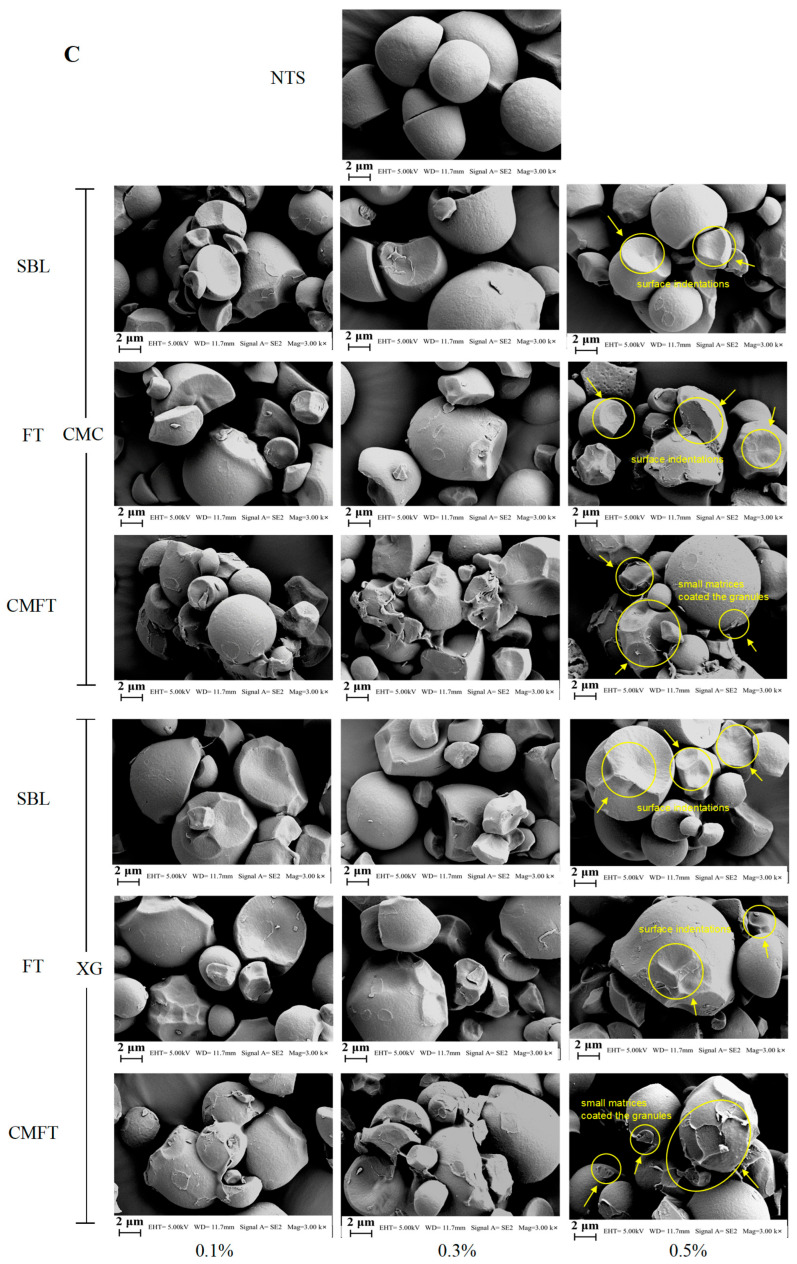
Soluble starch release (**A**), granular morphology and birefringence (**B**), and scanning electron micrographs (**C**) of tapioca starch (TS) and its composites with carboxymethyl cellulose (CMC) or xanthan gum (XG). The different letters within (**A**) indicate statistically significant differences (*p* < 0.05) among the various treatment and concentration combinations. NTS represents the native tapioca starch, SBL represents a simple blend of TS and hydrocolloid without treatment (control), FT represents freeze–thawing treatment, and CMFT represents critical melting combined with freeze–thawing treatment. All composites were prepared at hydrocolloid concentrations of 0.1%, 0.3%, or 0.5% (*w*/*w*, dry starch basis).

The soluble starch release during treatment is shown in Figure 1A. NTS showed the lowest iodine blue value, while both SBL and FT treatments slightly increased the blue value, indicating a minor release of soluble starch (mainly amylose). This may be attributed to physical stresses on the granules caused by the presence of hydrocolloids in SBL, and to the mechanical forces generated by ice-crystal formation during the FT. In contrast, CMFT treatment substantially increased the iodine blue value, from ~0.05 (NTS) and ~0.10 (SBL) to approximately 0.5–0.7 for both TS-CMC and TS-XG, particularly with increasing hydrocolloid addition. However, the granules retained their structural integrity after CMFT, as shown in Figure 1B,C, suggesting that CMFT predominantly disrupted amorphous regions and a part of ordered structures within starch granules, promoting disentanglement and solubilization of starch chains without causing serious granular disintegration. Such targeted structural weakening facilitated the release of soluble starch while preserving the granular framework, thereby more effectively promoting the reassociation of starch chains with hydrocolloids during subsequent FT. Critical melting may also promote the disentanglement of hydrocolloid chains (CMC or XG), enhancing their flexibility and accessibility for interaction with soluble starch. The subsequent freeze–thawing serves two functions: (i) ice crystal formation exerts localized mechanical stress on the already weakened granules, further promoting chain mobilization without complete disruption; (ii) the freeze-concentration effect locally increases the concentration of both released starch chains and hydrocolloids, thereby kinetically favoring interchain entanglement and network formation upon thawing. Moreover, ice-crystal formation during FT may further weaken the granular structure, facilitating additional release of mobile starch chains and contributing to the formation of an integrated composite matrix.

The granular morphology, birefringence, and surface microstructure of the composites are shown in Figure 1B,C. NTS exhibited typical morphology with clear birefringence and a smooth surface [24,25]. No significant alterations in granule morphology or birefringence were observed in either simple blends (SBL) or freeze–thaw (FT) treated samples, regardless of the hydrocolloid type (CMC or XG) or concentration (0.1–0.5%) (Figure 1B). Surface microstructure observation showed a similar trend (Figure 1C), revealing partially surface indentations and folding of the granules, especially at higher hydrocolloid concentrations. It might have resulted from the localized dehydration or surface adhesion of hydrocolloids during treatment, inducing physical stress on the granules [26,27]. For FT treatment, some small matrices coated on the granules, which might be aggregates of CMC or XG itself, since hydrocolloids are mainly located outside the granules via weak hydrogen bonds. These observations indicate that the interaction in SBL and FT occurred mainly on the surface rather than penetrating the granule interior.

In contrast, the CMFT treatment induced granule swelling and partial loss of birefringence (Figure 1B), indicating partial disruption of the internal granular structure, especially during the critical melting step [19]. These changes were clearly observed in both TS-CMC and TS-XG systems and became more evident with increasing hydrocolloid addition. As shown in Figure 1C, CMFT resulted in the formation of a large aggregated cluster composed of melted granules with a rough surface wrapped by the separated matrix. This matrix is attributed to the facilitated release of soluble starch chains following the partial disruption of double-helical order and hydrogen bond networks, which facilitate the interaction with hydrocolloids (CMC or XG) to form a matrix wrapping the swollen granules. Moreover, the weakened granule structure may facilitate more extensive interaction between hydrocolloids and starch chains within the granule interior. The subsequent FT further promoted interchain entanglement and network formation. The observed separated matrix differs from that observed on FT treatment, where the surface matrix consisted mainly of physically adhered hydrocolloid aggregates (Figure 1C). While the CMFT-induced matrix results from molecular entanglement and co-assembly of mobilized soluble starch chains with hydrocolloids.

Overall, the present observations indicate that the CMFT treatment promotes the formation of TS-CMC or TS-XG composites through a two-stage mechanism: partial granular melting with release of soluble starch chains. This process involves partial melting or weakening of the ordered structure, releasing soluble starch, and randomly disentangling CMC or XG chains, which alters the molecular arrangement and surface microstructure of the starch granules and may confer different functional properties to the composite.

### 3.2. Swelling Power, Water Solubility, Paste Transparency, and Granular Morphology Under Different Heating Conditions

**Figure 2:**
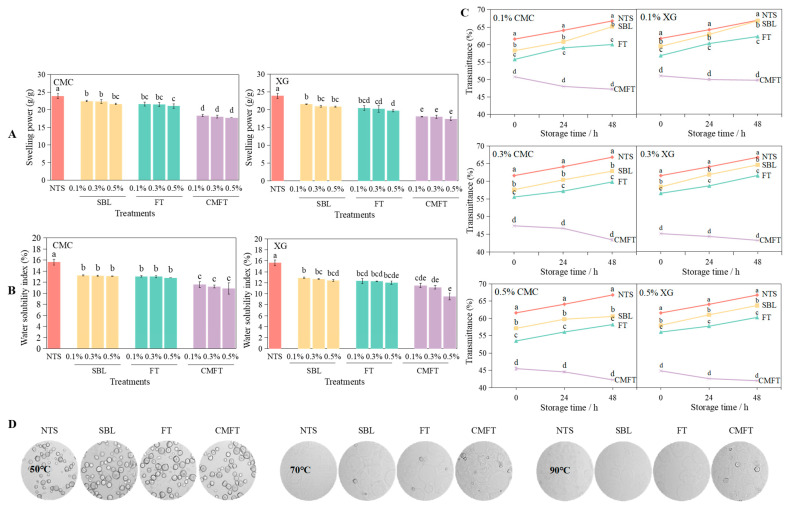
Swelling power (**A**), water solubility index (**B**), paste transmittance (**C**), and granular morphology (**D**) of tapioca starch (TS) and its composites with carboxymethyl cellulose (CMC) or xanthan gum (XG). (**D**) present granule morphology of samples heated at 50 °C, 70 °C, and 90 °C, respectively. All the error bars indicated the standard deviations (n = 3). The different letters within (**A**–**C**) indicate statistically significant differences (*p* < 0.05) among the various treatment and concentration combinations. NTS represents the native tapioca starch, SBL represents a simple blend of TS and hydrocolloid without treatment (control), FT represents freeze–thawing treatment, and CMFT represents critical melting combined with freeze–thawing treatment. All composites were prepared at hydrocolloid concentrations of 0.1%, 0.3%, or 0.5% (*w*/*w*, dry starch basis).

The swelling power (SP), water solubility (WSI), transmittance, and granular morphology of the composites are shown in Figure 2A–D. NTS exhibited the highest SP and WSI values, with granules undergoing initial swelling at 50 °C, extensive disruption at 70 °C, and complete gelatinization at 90 °C (Figure 2D). These changes indicate the high thermal sensitivity of NTS, which typically leads to the formation of a cohesive but mechanically weak and unstable paste network.

Compared with NTS, both SBL and FT treatments slightly reduced SP and WSI, suggesting that the presence of hydrocolloids (CMC and XG) can partially inhibit water penetration during heating. This effect was more evident with increasing hydrocolloid concentrations. However, this effect appeared limited, as SBL and FT samples still underwent extensive structural disruption at 70–90 °C, similar to NTS. These observations indicate that the starch-hydrocolloid interactions in SBL and FT composites are mainly on the granule surface via weak physical associations, thus difficult to substantially restrain thermal disintegration.

In contrast, CMFT-treated composites exhibited significantly lower SP and WSI values, indicating markedly enhanced interactions between starch and hydrocolloids (CMC or XG) and improved resistance to thermal disruption. Notably, after heating at 90 °C, CMFT granules showed swelling but largely retained their original morphology (Figure 2D), whereas extensive disintegration of granules was observed in SBL and FT, indicating that CMFT promoted TS-hydrocolloids network formation. The preserved granular integrity in CMFT samples suggested that the treatment promoted the formation of a cohesive TS-hydrocolloid network. These networks formed through the entanglement of released soluble starch with hydrocolloids, and the structural reorganization of the partially weakened granules themselves, effectively restricted water penetration and excessive swelling of granules, thereby preserving the structural integrity of the composite during thermal processing [10,16].

Paste transmittance results further supported the enhanced TS-hydrocolloids interactions (Figure 2C). All treated samples (SBL, FT, CMFT) initially exhibited lower transmittance than NTS, indicating immediate light scattering from restricted granule swelling or a dispersed phase. However, only CMFT paste showed consistently lower transmittance during the 48 h of storage period, suggesting the formation of a stable network (between TS and CMC or XG chains) that effectively prevented starch reassociation. In contrast, the transmittance of SBL and FT pastes increased significantly after 24 and 48 h of storage, approaching the values observed for the retrograde TS control, indicating a weak interaction in these systems. Due to these weak interactions, the initially intermixed starch with CMC or XG chains dissociated and reorganized into distinct starch-rich and hydrocolloid-rich domains during storage, reducing light scattering and increasing paste transmittance. The enhanced interactions and compact network formation during CMFT were further confirmed by the thermal properties and crystal structure analyses in the following sections.

### 3.3. Thermal Properties

The thermal properties of TS and its composites with CMC or XG are summarized in Table 1. The melting temperatures of To, Tp, and Tc for NTS were 65.52, 69.48, and 75.19 °C, respectively, with an enthalpy (ΔH) of 13.78 J/g. Both SBL and FT treatments slightly increased the gelatinization temperatures but decreased ΔH. These changes were attributed to competitive hydration between starch and hydrocolloids (CMC and XG), which partially limits water availability for gelatinization during blending, thereby reducing the enthalpic requirement for the phase transition. These results indicate that the intermolecular interactions introduced by SBL and FT are weak and limited, which makes it difficult to substantially alter the inherent structure or thermal behavior of starch.

In contrast, CMFT treatment substantially increased To, Tp, and Tc for both TS-CMC and TS-XG composites, particularly at higher hydrocolloid concentrations. For example, as shown in Table 1, CMFT raised To, Tp, and Tc of the TS-CMC composite (0.5% addition) to 69.55, 72.51, and 78.82 °C, and those of TS-XG to 69.13, 72.14, and 78.03 °C, respectively. This notable shift toward higher temperatures indicates the formation of a more thermally stable composite structure of TS-hydrocolloids. The partial disruption of starch granules during CMFT facilitated the release of soluble starch, which then interacts and entangles with hydrocolloid molecules to form a reinforced composite network that requires a higher temperature for dissociation [11,28,29]. Additionally, the CMC or XG can also act as a protective layer that competitively absorbs water from starch, thus further inhibiting starch melting [3,27,30,31].

Concurrently, CMFT caused a marked decrease in ΔH values, which dropped to approximately 9.9 J/g for both hydrocolloids at 0.5% addition, compared to 13.78 J/g for NTS. This decrease indicates a partial loss of original crystalline arrangement in starch during the CMFT process. Crucially, the CMFT-treated composites exhibited concurrent increases in gelatinization temperatures and decreases in ΔH. This contrasts with the typical behavior of thermally degraded starch, where a loss of crystalline order (lower ΔH) typically leads to a decrease in melting temperatures. This suggests that CMFT promotes a structural state in which residual ordered domains are fewer but more thermally stable, which could result from: (i) preferential disruption of less stable crystallites, leaving only those with higher melting temperatures; (ii) reduced water availability due to hydrocolloid interactions; and/or (iii) formation of a thermally stable amorphous network that physically restricts granular swelling. Therefore, these results suggest that CMFT is primarily an associative re-structuring process, rather than a simple disruptive one. Although chain rearrangement sacrifices some crystalline integrity (lowering ΔH), the interaction between leached starch chains and hydrocolloids promotes the formation of a more compact and thermally stable amorphous composite structure, resulting in an overall enhancement of thermal stability. Further results from SAXS and FTIR support the interpretation that chain reassociation between starch and hydrocolloids contributes to this phenomenon.

Overall, the DSC data in Table 1 showed that CMFT significantly enhances the interaction between TS and hydrocolloids, with increased gelatinization temperatures and reduced ΔH. These findings indicate that CMFT facilitates the transformation of the TS-hydrocolloid system from a simple physical blend into a compact and chain-entangled composite with enhanced thermal stability.

### 3.4. Long-Range Ordered Structure

**Figure 3:**
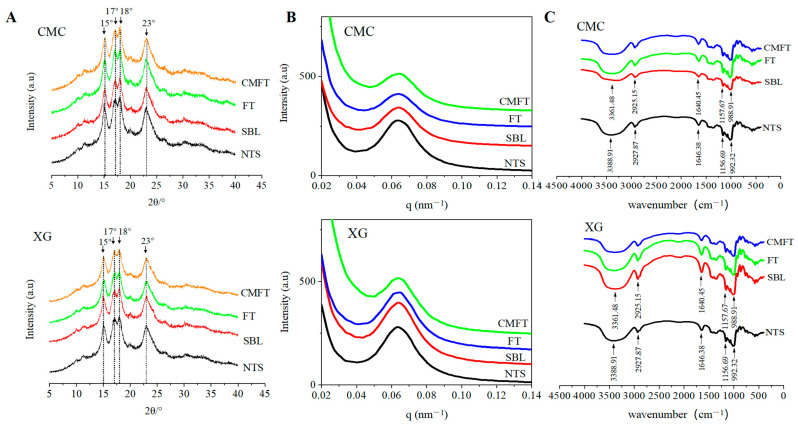
X-ray diffraction patterns (**A**), Small angle X-ray scatterings (**B**), and FTIR spectrum (**C**) of tapioca starch (TS) and its composites with carboxymethyl cellulose (CMC) or xanthan gum (XG). NTS represents the native tapioca starch, SBL represents a simple blend of TS and hydrocolloid without treatment (control), FT represents freeze–thawing treatment, and CMFT represents critical melting combined with freeze–thawing treatment.

The long-range ordered structure and the XRD patterns of TS and its composites with CMC or XG are presented in Figure 3A and Table 2. NTS exhibited a characteristic A-type XRD pattern, characterized by strong diffraction peaks at 15°, 17°, 18°, and 23°. The calculated crystallinity parameters, including CR% (crystalline region), SR% (subcrystalline region), and RC% (relative crystallinity), were 12.36, 13.52, and 25.88%, respectively [24].

SBL did not change the A-type crystal pattern of the composite, but slightly reduced the overall intensity of diffraction peaks, with the CR%, SR%, and RC% decreased to approximately 11.00, 13.00, and 24.00%, respectively. The effect was slightly more pronounced at higher hydrocolloid concentrations. These changes suggest that the presence of CMC or XG primarily exerts superficial interference, which mildly affects starch chain packing but makes it difficult to disrupt the long-range crystalline order within the granule interior. FT treatment induced a further decrease in crystallinity parameters, with CR%, SR%, and RC% to approximately 11.00, 12.00, and 23.00%, respectively. The A-type diffraction pattern persisted, indicating that FT alone does not alter the crystal type of the composite. The additional reduction in crystallinity observed in FT compared to SBL was likely attributable to the physical stress produced by ice crystal formation during freeze–thaw, which may locally disrupt the peripheral granular organization of starch [19].

As expected, CMFT led to substantial disruption of the long-range ordered structure. This was evidenced by a marked decrease in CR%, SR%, and RC% to approximately 9.00%, 11.00%, and 20.00%, respectively, and DCL% ranging from 17% to 22%, indicating a marked loss of long-range crystalline order and a transition towards a more disordered or amorphous state. This disruption of starch crystallinity is a fundamental prerequisite for enhanced functional properties. The breakdown of the crystalline structure released disordered starch chains, which became available for extensive molecular interaction and entanglement with hydrocolloid chains (CMC or XG) to form a compact composite matrix. This led to the formation of a composite matrix characterized by a reinforced amorphous network, derived from the interacting biopolymer chains, coexisting with residual, reorganized crystalline domains.

Therefore, the XRD data indicated that the substantial reduction in crystallinity and the increase in structural disorder induced by CMFT were crucial for enhancing TS-hydrocolloids (CMC or XG) interactions. The structural modifications induced by CMFT are consistent with observations in other starch systems subjected to controlled thermal treatments. Zhang et al. [19] reported similar reductions in crystallinity in potato starch after critical melting and freeze–thawing treatments, while Wang et al. [29] observed structural reorganization in rice starch during retrogradation. Unlike SBL or FT, CMFT could uniquely dismantle the crystalline framework of starch to promote chain reassociation with hydrocolloids. This structural reorganization provided the mechanistic basis for the improved functional properties, particularly the improved thermal stability discussed in Section 3.3. Compared with other physical modification approaches, CMFT offers distinct advantages. Unlike annealing treatment, which typically increases starch crystallinity and thermal stability without releasing significant soluble starch [3], CMFT induced partial disruption of crystalline regions while releasing soluble chains for interaction with hydrocolloids. This distinguishes CMFT from ultrasonic treatment, which primarily affects surface morphology, or from heat-moisture treatment, which mainly alters granular crystallinity without promoting extensive chain release.

### 3.5. Lamellar Structure

The lamellar structure characteristics of TS and its composites with CMC or XG are presented in Figure 3B and Table 3, respectively. NTS exhibited a well-defined scattering peak at q = 0.64 nm^−1^, and the dBragg, Imax, and Ap were 9.92 nm, 234.22, and 4.55, respectively, indicating a highly ordered semi-crystalline lamellar structure with strong electron density contrast between crystalline and amorphous regions [32]. SBL slightly reduced the Imax and Ap values without significantly altering q or dBragg, for both TS-CMC and TS-XG composites. It was attributed to the superficial interference of hydrocolloids through weak hydrogen bonding and steric hindrance, which mildly disrupts the ordered packing of double helices within the lamellae. FT induced a further decrease in both Imax and Ap, while q and dBragg showed no significant decrease. This indicated that the lamellar repeat distance was preserved despite the reduced scattering contrast during FT, which is attributed to the physical stress generated by ice crystal formation during freezing, enhancing the disruptive effect of hydrocolloids on the lamellar structure.

CMFT treatment induced a significant disruption of the lamellar structure. As shown in Table 3, CMFT markedly decreased Imax and Ap to approximately 157–172 and 2.2–2.6, respectively, with further reductions observed at higher hydrocolloid concentrations. These changes indicated a loss of electron density contrast and disordering of structure between the crystalline and amorphous lamellar phases [1,2,4]. The disruption was primarily due to the extensive breakage of intra- and intermolecular hydrogen bonds within starch granules during the CMFT process, weakening the structural integrity of both the crystalline and amorphous regions.

However, the dBragg values for CMFT-treated composites remained within the range of 9.73–9.78 nm^−1^, similar to those of NTS and other treatments. This indicated that although CMFT disrupted the internal order of the lamellae, the average periodic distance between residual lamellar structures was not significantly altered. These changes also suggested that the disruption occurred primarily within the lamellae rather than through large-scale swelling, delamination, or complete collapse of the lamellar architecture [13,20]. The substantial decrease in Imax and Ap, without the loss of lamellar periodicity, could be attributed to two contributing factors. Firstly, partial disruption of the crystalline lamellae (consistent with the decreased RC% in Section 3.4) decreased the electron density of the crystalline phase. Secondly, CMFT promoted extensive chain mobilization and intermixing between TS and hydrocolloids within the existing lamellar structure. The soluble starch chains, particularly amylose, were released from the crystalline lamellae and became entangled with hydrocolloid chains in the amorphous lamellae. This chain reassociation and redistribution thus reduced the electron density contrast, as the amorphous lamellae became enriched with hydrocolloid chains while the crystalline lamellae became partially disrupted and less perfectly ordered.

### 3.6. Short-Range Ordered Structure

The FTIR spectra and corresponding absorbance ratios of TS and its composites are presented in Figure 3C and Table 3. No new absorption peaks were observed after hydrocolloid addition or physical treatment, indicating that no new functional groups and covalent bonds were formed between TS and hydrocolloids [33]. Both SBL and FT treatments slightly decreased R1047/1022 from 0.91 (NTS) to 0.86–0.89 and increased R1022/995 from 1.06 (NTS) to 1.09–1.13, respectively. Compared with SBL and FT, CMFT significantly decreased R1047/1022 to approximately 0.83 and increased R1022/995 to approximately 1.18, respectively. These changes became more significant with increasing hydrocolloid addition. The substantial decrease in R1047/1022 indicates the disruption of double-helical integrity, which agrees well with the reduced relative crystallinity (XRD) and loss of lamellar electron density contrast (SAXS) discussed above. The marked increase in R1022/995 indicates the formation of a stronger hydrogen-bonding environment, suggesting the replacement of some starch-starch hydrogen bonds by starch-hydrocolloid interactions. These results suggest that CMFT promotes chain mobilization and interengagement between TS and hydrocolloids within the amorphous regions, thereby facilitating the formation of a reorganized composite matrix.

### 3.7. Pasting Properties

**Figure 4:**
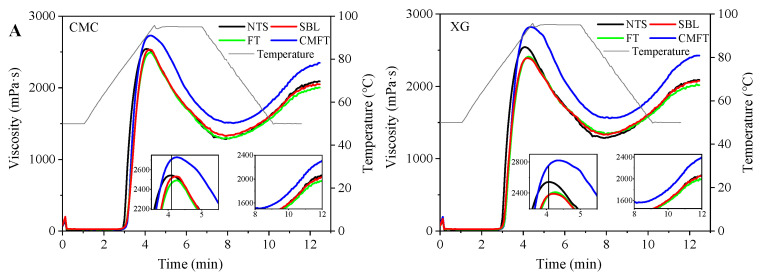

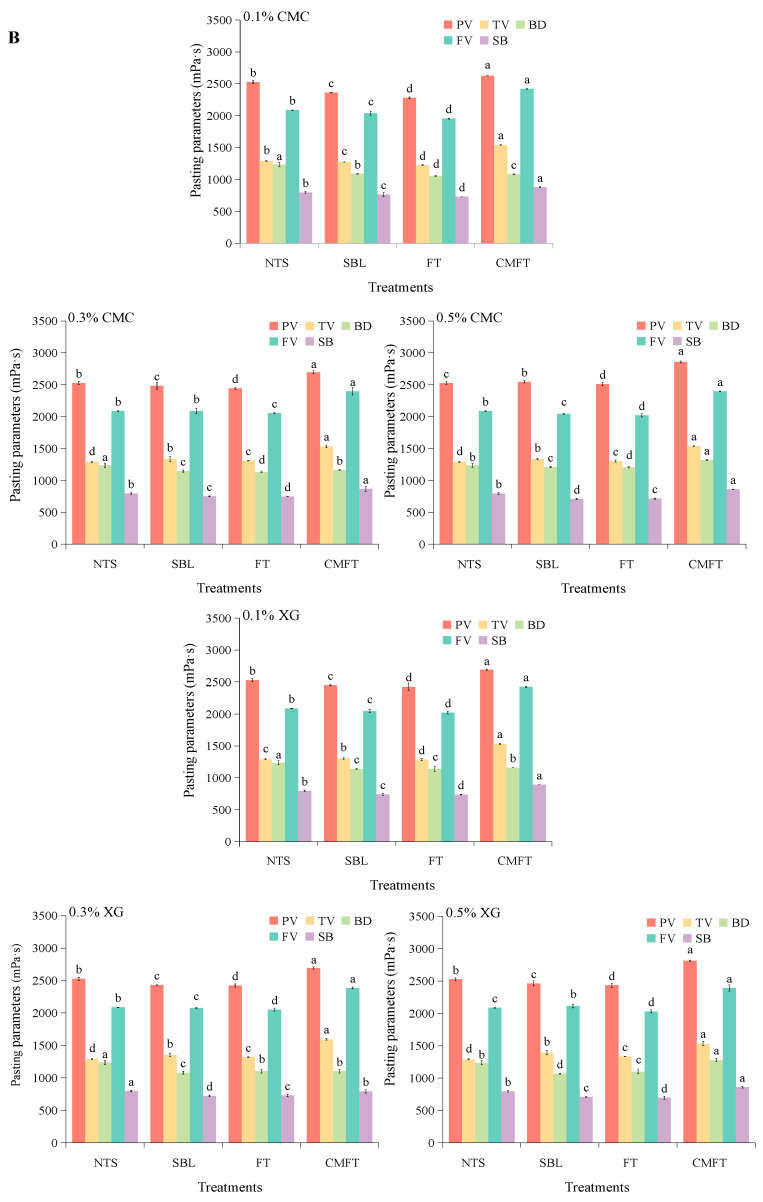
Pasting curves (**A**) and pasting parameters (**B**) of tapioca starch (TS) and its composites with carboxymethyl cellulose (CMC) or xanthan gum (XG). (**A**) shows representative images of the sample with 0.5% hydrocolloids addition. The different letters within (**B**) indicate statistically significant differences (*p* < 0.05) among the various treatment and concentration combinations. NTS represents the native tapioca starch, SBL represents a simple blend of TS and hydrocolloid without treatment (control), FT represents freeze–thawing treatment, and CMFT represents critical melting combined with freeze–thawing treatment. All composites were prepared at hydrocolloid concentrations of 0.1%, 0.3%, or 0.5% (*w*/*w*, dry starch basis).

The pasting curve and viscosity parameters of TS and its composites with CMC or XG are presented in Figure 4A,B. NTS exhibited a typical pasting curve with PV of approximately 2500 mPa·s, TV of 1200 mPa·s, FV of 2000 mPa·s, and BD of 1200 mPa·s. For both CMC and XG composites, SBL and FT treatments did not alter the overall shape of the pasting curve, with a minor decrease in pasting viscosity. This was attributed to the competitive hydration of hydrocolloids, which partially limits water availability for granule swelling and weakly coats the granule surface. These limited interactions between TS and hydrocolloids were attributed to the surface adhesion of hydrocolloids and their competition for water molecules, which mildly restricts initial granular swelling [5,19].

Compared with NTS, SBL, and FT, CMFT significantly increased the PV, TV, and FV to approximately 2700, 1530, and 2400 mPa·s, respectively. Moreover, peak time and pasting temperature increased from 4.16 min and 73.78 °C (NTS) to ~4.37 min and ~74.5 °C, indicating increased shear stability and thermal stability of the composite. The partial disruption of crystalline and lamellar order releases soluble starch chains that entangle with hydrocolloids, forming a reinforced amorphous matrix. These pre-existing networks may partially restrict granular swelling during heating (which would tend to lower PV); however, the overall effect of CMFT on PV is governed by multiple competing factors. The released soluble starch chains entangled with hydrocolloids form a continuous viscoelastic network that directly contributes to the viscosity of the paste. Additionally, the rough granular surface and the surrounding matrix observed in SEM (Figure 1C) increase intergranular friction and flow resistance during pasting. These positive contributions to viscosity outweigh the moderate suppression of swelling, resulting in a net increase in PV. The same pre-existing networks also stabilize the granules against shear, as reflected by the reduced BD, and promote chain reassociation upon cooling to develop higher FV and SB. These pasting characteristics are similar to those observed for chemically cross-linked starches and are consistent with the interpretation that CMFT transforms the weak physical blend into a structurally integrated composite with enhanced thermal stability and shear resistance, although direct evidence of covalent crosslinking is not provided and the observed effects likely arise from physical entanglement and hydrogen-bonding interactions.

### 3.8. Dropping Properties and Viscoelastic Tensile Properties of the Paste

**Figure 5:**
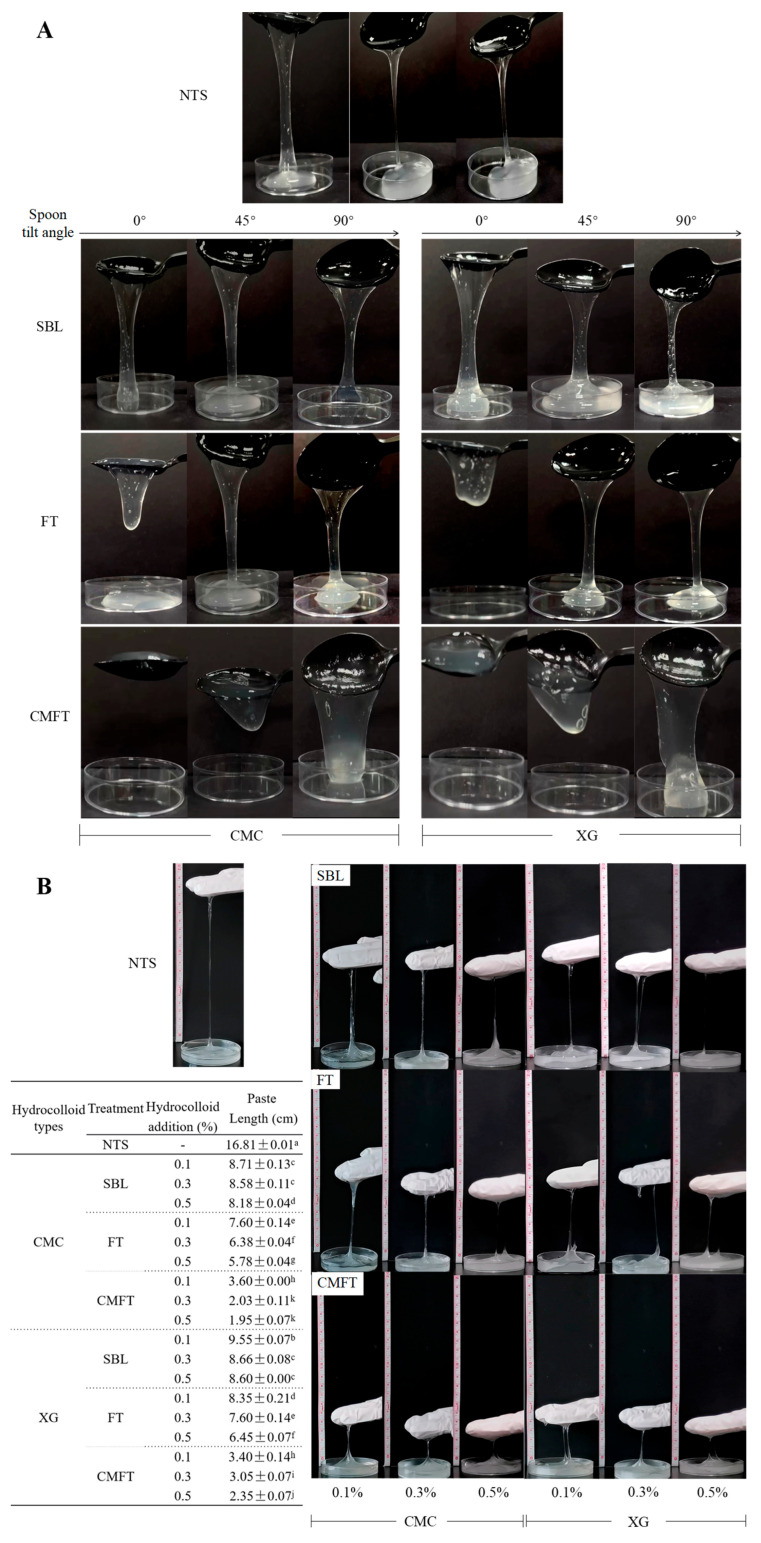

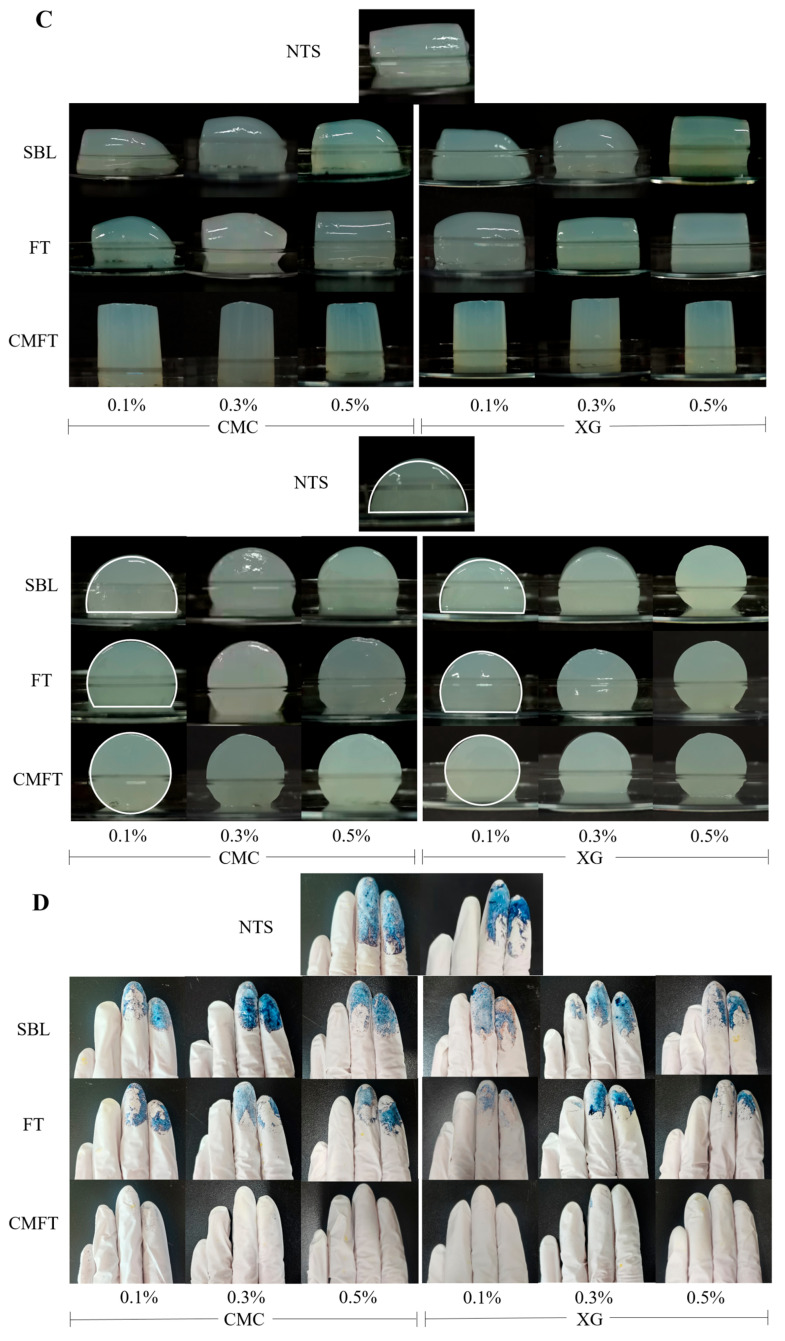
Paste dropping properties (**A**), paste length (**B**), gel morphology (**C**) and apparent adhesion properties (**D**) of tapioca starch (TS) and its composites with carboxymethyl cellulose (CMC) or xanthan gum (XG). The different letters within (**B**) indicate statistically significant differences (*p* < 0.05) among the various treatment and concentration combinations. NTS represents the native tapioca starch, SBL represents a simple blend of TS and hydrocolloid without treatment (control), FT represents freeze–thawing treatment, and CMFT represents critical melting combined with freeze–thawing treatment. All composites were prepared at hydrocolloid concentrations of 0.1%, 0.3%, or 0.5% (*w*/*w*, dry starch basis).

The dropping behavior and viscoelastic tensile properties of starch paste are critical physical attributes that determine its suitability for food applications requiring cohesive and structured textures, such as sauces or jellies. As shown in Figure 5A,B, NTS paste exhibited a thin, continuous, and weak texture, stretching to 16.81 cm before breaking. This is attributed to the high swelling power and low amylose content of TS, which results in a weakly entangled network with limited cohesive strength and an undesirable sticky mouthfeel. Compared with NTS, the SBL and FT treatments decreased the paste length to approximately 7–8 cm, especially at higher hydrocolloid concentrations, indicating that CMC or XG can physically enhance paste cohesion through surface adhesion and weak intermolecular interactions [14,16]. However, the resulting paste texture remained relatively weak and sticky.

As shown in Figure 5A,B, CMFT significantly decreased the paste length from 16.81 cm (TS) to approximately 2–3 cm, with further reductions observed at higher hydrocolloid additions. This suggests a transition from a ductile, extensible paste to a more brittle, cohesive network, which may be attributable to the enhanced molecular interactions between starch and hydrocolloids. This is in contrast to the weak intermolecular associations and poor texture inherent to NTS paste. Furthermore, when the spoon tilt angle was varied from 0° to 45° and 90°, CMFT-treated pastes consistently maintained a compact and cohesive network. Similarly, as shown in Figure 5B, NTS paste adhered extensively to the fingertip and formed a long, stringy filament upon withdrawal, indicating poor network strength and high adhesiveness. In contrast, CMFT-treated pastes exhibited short, clean detachment, and the paste surface rapidly recovered flatness after finger removal, indicating enhanced elasticity and network integrity. Therefore, CMFT-treated composites exhibited a short, cohesive, and structured paste network, in contrast to the weak, elongated, and sticky NTS paste. This improvement is attributed to the partial disruption of crystalline order and enhanced chain entanglement between released starch and hydrocolloids.

### 3.9. Gel Morphology and Apparent Surface Adhesion Properties

Gel morphology and apparent surface adhesion properties of TS and its composites with CMC or XG are presented in Figure 5C,D. NTS gel exhibited a weak and poorly structured morphology, and was unable to maintain an upright position on a flat surface. After gentle rolling the NTS gel over a distance of 10 cm observed extensive gel tissue adhering to the glove surface, and showed an intense iodine staining on the glove surface, indicating a weak internal network with poor cohesiveness and high surface adhesiveness [22]. Gel prepared by SBL and FT treatments showed improved overall morphology and lighter iodine staining compared with NTS. However, these gels were still unable to maintain a stable upright position, and some gel tissue still adhered to the glove after rolling, indicating that the interactions between TS and hydrocolloids remained limited. Even at 0.5% hydrocolloid addition, the bottom contact area of gels with the table surface remained extensive, reflecting a persistently soft texture and limited network development.

In contrast, CMFT prepared gels exhibited substantial improvements in gel texture, forming a well-structured morphology with a smooth surface, and maintained a stable upright position, even at the minimal addition of 0.1% CMC or XG. The bottom profile showed a markedly reduced contact area with the flat surface, suggesting the formation of a more cohesive gel structure. Notably, after rolling, these gels showed no residual gel tissue or iodine staining, indicating a substantially improved gel network. These observations suggest that CMFT promotes extensive interchain entanglement between starch and hydrocolloids, forming a stable and well-defined internal network with enhanced cohesiveness and reduced surface adhesiveness.

### 3.10. Gel Texture Properties and Microstructure

**Figure 6:**
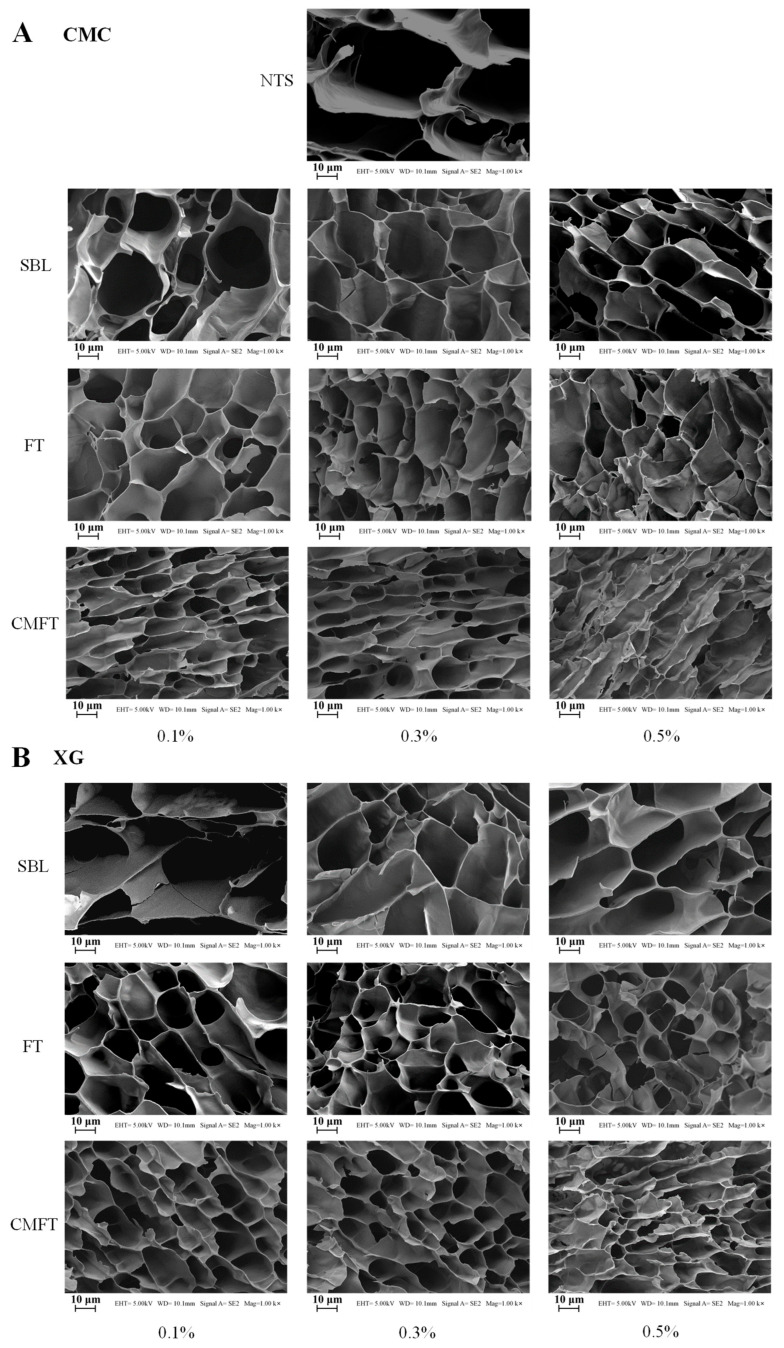
Gel microstructure of tapioca starch (TS) and its composites with carboxymethyl cellulose (CMC) (**A**) or xanthan gum (XG) (**B**). NTS represents the native tapioca starch, SBL represents a simple blend of TS and hydrocolloid without treatment (control), FT represents freeze–thawing treatment, and CMFT represents critical melting combined with freeze–thawing treatment. All composites were prepared at hydrocolloid concentrations of 0.1%, 0.3%, or 0.5% (*w*/*w*, dry starch basis).

The gel texture properties and corresponding microstructures of TS and its composites with CMC or XG are presented in Table 4 and Figure 6 NTS gel exhibited poor texture characteristics, with low hardness (45.16 gf), springiness (0.78), and chewiness (33.75 gf). As shown in Figure 6, the microstructure of NTS gel was characterized by uneven pore size, thin pore walls, with a fragmented and discontinuous architecture. These features collectively indicate a weak and poorly structured gel network with limited mechanical integrity. Compared with NTS, the SBL and FT treatments slightly increased the gel texture properties, and the pore sizes of microstructure became smaller and more uniform.

In contrast, gel prepared by CMFT exhibited significantly improved texture properties, with increased hardness, springiness, and chewiness from 45.16, 0.78, and 33.75 (NTS) to approximately 130, 0.90, and 100 for both composite systems. These substantial increases indicate a marked improvement in gel mechanical strength and texture characteristics. Correspondingly, CMFT-prepared gels exhibited a dense, well-organized microstructure with regular, uniform pores, indicating a more compact and stable gel network. The enhanced gel properties are attributed to the synergistic interactions between TS and hydrocolloids promoted by CMFT. Partial melting of the ordered starch structure during heating facilitates the release of amylose chains, which can then effectively interact with CMC or XG to form a cohesive matrix, thus filling the interstitial spaces within the gel and promoting the formation of a sheet-like structure. Furthermore, CMFT treatment led to the formation of a uniform and continuous phase between TS and hydrocolloids, contributing to a denser and significantly more stable gel network [1,4,13]. The dense, uniform pore structure in CMFT gels (Figure 6) directly explains the ~3-fold increase in hardness and chewiness (Table 4), as the continuous sheet-like network resists deformation more effectively.

### 3.11. In Vitro Digestibility

**Figure 7:**
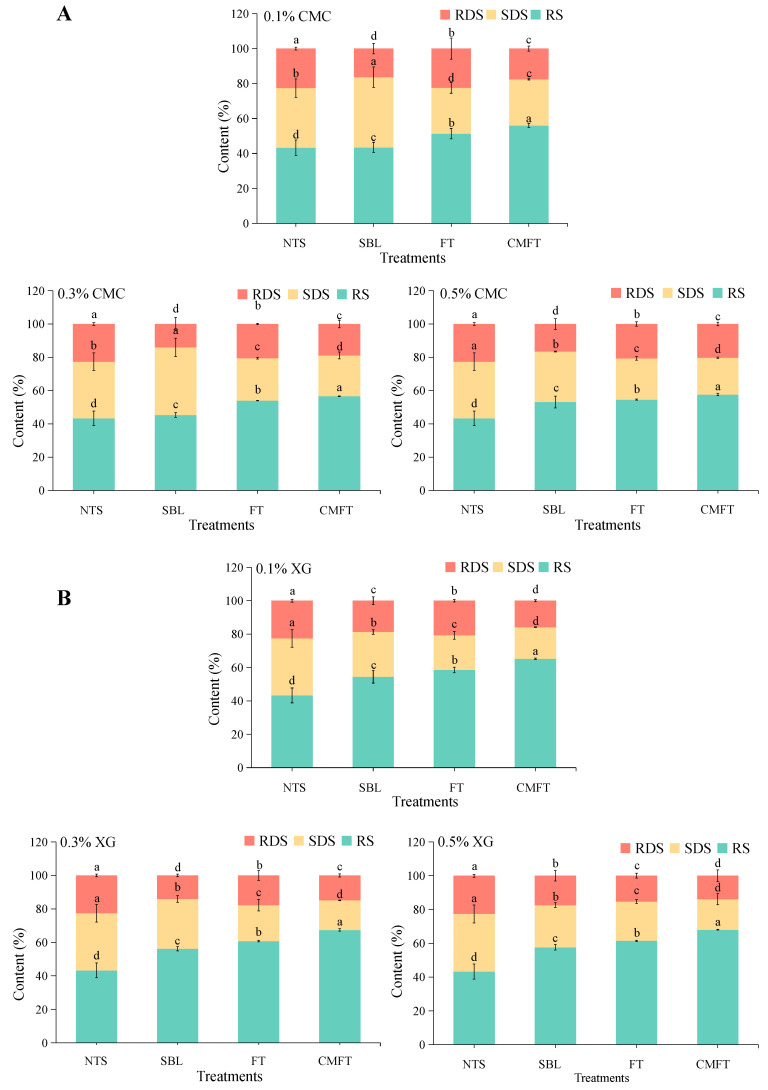
In vitro digestibility of tapioca starch (TS) and its composites with carboxymethyl cellulose (CMC) (**A**) or xanthan gum (XG) (**B**). The different letters within Figure 7 indicate statistically significant differences (*p* < 0.05) among the various treatment and concentration combinations. NTS represents the native tapioca starch, SBL represents a simple blend of TS and hydrocolloid without treatment (control), FT represents freeze–thawing treatment, and CMFT represents critical melting combined with freeze–thawing treatment. All composites were prepared at hydrocolloid concentrations of 0.1%, 0.3%, or 0.5% (*w*/*w*, dry starch basis).

The in vitro digestibility of TS and its composites with CMC or XG is presented in Figure 7. The RDS, SDS, and RS content of NTS were 22.61%, 34.11%, and 43.28%, respectively. Compared with NTS, SBL and FT decreased the RDS and SDS while increasing the RS. This is attributed to the physical barrier formed by hydrocolloids adhering to the granule surface, which limits enzyme access to the starch substrate. Additionally, the increased viscosity of the composite system may further impede enzyme diffusion and penetration [5,10].

Compared with NTS, CMFT decreased RDS content from 22.61% to approximately 18%, while substantially increasing RS content from 43.28% to ~60%. This is attributable to the structural reorganization induced by CMFT, as characterized in previous sections. Partial disruption of crystalline order releases starch chains that become extensively entangled with CMC or XG, forming a dense, cohesive amorphous network. This reinforced matrix physically inhibits enzyme penetration and diffusion, thereby reducing the accessible surface area of the composite for hydrolysis. The substantial increase in RS content indicates that this network effectively encapsulates starch fractions, rendering them resistant to enzymatic digestion. Notably, CMFT treatment increased RS content for both CMC and XG composites, with values ranging from approximately 56% to 58% for CMC and from 65% to 68% for XG at the same 0.1–0.5% addition levels. This indicates that the enhanced entanglement is broadly achievable across hydrocolloid types, but the magnitude of the effect differs between the two. XG-containing composites consistently exhibited higher RS values than their CMC counterparts under identical CMFT conditions. This difference may be attributed to the distinct conformational and hydration properties of XG. Its ordered helical structure and higher solution viscosity could form a more effective physical barrier to enzyme diffusion compared to the linear, more flexible CMC chains. These observations suggest that while CMFT effectively promotes starch-hydrocolloid interactions for both hydrocolloids, the degree of digestibility reduction is modulated by hydrocolloid specific factors. The partial disruption of starch crystalline structure during CMFT releases soluble starch chains that serve as primary building blocks of the composite network, while hydrocolloids act as auxiliary crosslinkers that reinforce the starch-dominated matrix. Consequently, the CMFT-induced structural reorganization of starch plays a dominant role, but hydrocolloid structure still exerts a secondary, non-negligible influence on the final functional properties. This suggests that CMFT may serve as a promising strategy for preparing starch-hydrocolloid composites with enhanced resistant starch content, although the optimal hydrocolloid choice may depend on the specific target application.

## 4. Conclusions

This study demonstrates that CMFT effectively enhances the interactions between TS and hydrocolloids (CMC or XG) by promoting structural weakening and chain reassociation, as evidenced by multiple structural and functional analyses. The partial thermal disruption of the native crystalline order releases soluble starch chains, facilitating their reassociation and entanglement with hydrocolloids during CMFT, thereby forming a cohesive and structurally integrated composite matrix. The resulting structural reorganization significantly enhances thermal and shear stability, as well as gel texture and paste cohesiveness. CMFT transforms TS paste from a weak, elongated structure into a cohesive, structurally integrated composite suitable for clean-label applications such as thickeners, stabilizers, and gelling agents in food products. Furthermore, the formation of this dense, entangled matrix effectively reduces enzymatic digestibility, significantly increasing the resistant starch content from 43.28% to approximately 60%. Notably, CMFT treatment showed comparable enhancements in pasting, gel, and digestive properties for both CMC and XG, suggesting that the strategy may be broadly applicable across hydrocolloids with different structural features. The observed changes, including reduced crystallinity, increased gelatinization temperatures, improved paste and gel properties, and increased resistant starch contents, are consistent with the proposed mechanism of structural weakening followed by chain reassociation. These findings provide a theoretical basis and a practical processing strategy for designing functional starch-based ingredients with tailored properties for food and industrial applications. However, further research is needed to verify the applicability of CMFT to other starch sources and hydrocolloids, and to evaluate its performance under real food processing conditions and in vivo nutritional outcomes.

## Figures and Tables

**Table 1:** Thermal properties of tapioca starch (TS) and its composites with carboxymethyl cellulose (CMC) or xanthan gum (XG).

Hydrocolloid Types	Treatments	Hydrocolloid Addition (%)	Thermal Temperatures			ΔH/(J/g)
			To/°C	Tp/°C	Tc/°C	
-	NTS	-	65.52 ± 1.58 ^e^	69.48 ± 2.20 ^b^	75.19 ± 4.04 ^b^	13.78 ± 1.68 ^a^
CMC	SBL	0.1	66.73 ± 0.19 ^cde^	71.31 ± 0.15 ^ab^	77.53 ± 0.02 ^ab^	13.72 ± 0.03 ^ab^
		0.3	66.27 ± 0.04 ^de^	70.66 ± 0.19 ^ab^	77.57 ± 0.28 ^ab^	13.46 ± 0.11 ^ab^
		0.5	66.85 ± 0.08 ^cde^	71.64 ± 0.07 ^ab^	78.66 ± 0.01 ^ab^	13.27 ± 0.12 ^ab^
	FT	0.1	66.22 ± 0.49 ^de^	71.04 ± 0.20 ^ab^	78.98 ± 0.14 ^ab^	12.46 ± 0.45 ^abc^
		0.3	66.24 ± 1.05 ^de^	72.06 ± 3.51 ^ab^	79.78 ± 5.94 ^a^	11.55 ± 1.51 ^abcd^
		0.5	66.70 ± 0.44 ^cde^	71.77 ± 1.31 ^ab^	78.12 ± 0.45 ^ab^	11.48 ± 1.12 ^abcd^
	CMFT	0.1	67.61 ± 1.85 ^abcde^	71.22 ± 0.95 ^ab^	77.40 ± 0.45 ^ab^	11.05 ± 0.45 ^abcd^
		0.3	68.94 ± 0.93 ^abc^	72.26 ± 0.77 ^a^	78.37 ± 0.82 ^ab^	9.88 ± 2.64 ^cd^
		0.5	69.55 ± 0.78 ^a^	72.51 ± 0.08 ^a^	78.82 ± 0.31 ^ab^	8.64 ± 0.08 ^d^
XG	SBL	0.1	66.56 ± 0.04 ^de^	71.48 ± 0.23 ^ab^	78.37 ± 0.36 ^ab^	12.37 ± 0.03 ^abc^
		0.3	66.63 ± 0.29 ^de^	71.15 ± 0.08 ^ab^	78.80 ± 0.46 ^ab^	12.36 ± 0.66 ^abc^
		0.5	66.80 ± 0.14 ^cde^	71.33 ± 0.13 ^ab^	79.10 ± 0.69 ^ab^	12.33 ± 0.04 ^abc^
	FT	0.1	67.97 ± 1.25 ^abcd^	71.97 ± 0.49 ^ab^	78.45 ± 0.07 ^ab^	11.88 ± 1.53 ^abc^
		0.3	66.29 ± 0.01 ^de^	70.45 ± 0.35 ^ab^	78.84 ± 0.33 ^ab^	11.37 ± 0.22 ^abcd^
		0.5	67.31 ± 0.25 ^bcde^	71.45 ± 0.25 ^ab^	78.49 ± 0.23 ^ab^	11.34 ± 0.06 ^abcd^
	CMFT	0.1	67.88 ± 2.03 ^abcd^	71.21 ± 0.83 ^ab^	77.57 ± 0.52 ^ab^	10.96 ± 0.72 ^abcd^
		0.3	68.16 ± 1.23 ^abcd^	71.82 ± 0.50 ^ab^	77.96 ± 0.22 ^ab^	10.65 ± 3.39 ^bcd^
		0.5	69.13 ± 0.49 ^ab^	72.14 ± 0.51 ^a^	78.03 ± 0.02 ^ab^	9.60 ± 1.44 ^cd^

All data represent the mean ± standard deviation of triplicate measurements. Different superscript letters within the same column indicate statistically significant differences (*p* < 0.05) among the various treatment and concentration combinations. NTS represents the native tapioca starch, SBL represents a simple blend of TS and hydrocolloid without treatment (control), FT represents freeze–thawing treatment, and CMFT represents critical melting combined with freeze–thawing treatment. All composites were prepared at hydrocolloid concentrations of 0.1%, 0.3%, or 0.5% (*w*/*w*, dry starch basis).

**Table 2:** Long ordered structure of tapioca starch (TS) and its composites with carboxymethyl cellulose (CMC) or xanthan gum (XG).

Hydrocolloid Types	Treatments	Hydrocolloid Addition (%)	CR (%)	SR (%)	RC (%)	DCL (%)	Diffraction Angle/Interplanar Spacing (Å)			
							15	17	18	23
-	NTS	-	12.36 ± 0.41 ^a^	13.52 ± 0.23 ^a^	25.88 ± 0.18 ^a^	-	15.05 (5.88)	17.11 (5.17)	18.01 (4.92)	23.03 (3.86)
CMC	SBL	0.1	11.13 ± 0.18 ^b^	13.30 ± 0.28 ^a^	24.42 ± 0.45 ^b^	5.62	14.95 (5.92)	16.96 (5.22)	18.01 (4.92)	23.14 (3.84)
		0.3	11.11 ± 0.15 ^b^	13.20 ± 0.28 ^ab^	24.31 ± 0.13 ^bc^	6.07	15.09 (5.87)	17.08 (5.19)	18.03 (4.90)	23.03 (3.86)
		0.5	11.05 ± 0.21 ^b^	13.00 ± 0.00 ^abc^	24.05 ± 0.21 ^bc^	7.05	14.92 (5.93)	17.03 (5.20)	17.94 (4.94)	22.94 (3.87)
	FT	0.1	11.11 ± 0.28 ^b^	12.75 ± 0.07 ^bc^	23.86 ± 0.35 ^bc^	7.81	15.13 (5.85)	17.08 (5.19)	18.07 (4.91)	23.08 (3.85)
		0.3	11.07 ± 0.03 ^b^	12.73 ± 0.04 ^bc^	23.80 ± 0.01 ^bc^	8.04	15.05 (5.88)	17.12 (5.18)	18.11 (4.89)	23.12 (3.84)
		0.5	10.95 ± 0.35 ^b^	12.50 ± 0.14 ^cd^	23.45 ± 0.21 ^cd^	9.37	14.96 (5.92)	17.21 (5.15)	17.98 (4.93)	22.81 (3.89)
	CMFT	0.1	9.84 ± 0.19 ^c^	11.67 ± 0.09 ^e^	21.50 ± 0.28 ^fg^	16.91	14.87 (5.95)	16.86 (5.25)	18.20 (4.87)	22.86 (3.89)
		0.3	9.80 ± 0.28 ^c^	11.60 ± 0.42 ^e^	21.40 ± 0.14 ^g^	17.29	15.22 (5.82)	16.82 (5.27)	17.85 (4.96)	22.90 (3.88)
		0.5	8.55 ± 0.64 ^d^	11.50 ± 0.57 ^e^	20.05 ± 0.07 ^h^	22.51	15.26 (5.80)	16.95 (5.23)	18.24 (4.85)	23.20 (3.83)
XG	SBL	0.1	11.28 ± 0.32 ^b^	13.30 ± 0.14 ^a^	24.58 ± 0.18 ^b^	5.02	15.04 (5.88)	17.03 (5.20)	18.02 (4.91)	23.03 (3.86)
		0.3	11.25 ± 0.07 ^b^	13.00 ± 0.14 ^abc^	24.25 ± 0.21 ^bc^	6.28	15.17 (5.83)	16.98 (5.21)	18.06 (4.90)	22.99 (3.86)
		0.5	11.20 ± 0.85 ^b^	12.20 ± 0.28 ^d^	23.40 ± 1.13 ^cd^	9.57	14.96 (5.91)	17.07 (5.18)	17.98 (4.92)	23.07 (3.85)
	FT	0.1	11.15 ± 0.35 ^b^	11.55 ± 0.21 ^e^	22.70 ± 0.57 ^de^	12.27	15.00 (5.90)	17.21 (5.15)	17.85 (4.96)	23.07 (3.85)
		0.3	10.95 ± 0.64 ^b^	11.40 ± 0.14 ^e^	22.35 ± 0.49 ^ef^	13.62	15.21 (5.81)	16.90 (5.24)	17.93 (4.94)	23.24 (3.82)
		0.5	10.78 ± 0.11 ^b^	11.38 ± 0.11 ^e^	22.15 ± 0.21 ^efg^	14.40	15.09 (5.87)	17.16 (5.16)	18.15 (4.88)	23.20 (3.82)
	CMFT	0.1	9.25 ± 0.64 ^cd^	11.30 ± 0.00 ^e^	20.55 ± 0.64 ^h^	20.58	14.83 (5.96)	17.2 (5.14)	18.62 (4.91)	22.90 (3.87)
		0.3	9.13 ± 0.53 ^cd^	11.23 ± 0.32 ^e^	20.35 ± 0.21 ^h^	21.35	15.34 (5.76)	16.81 (5.26)	17.72 (5.00)	22.94 (3.87)
		0.5	8.97 ± 0.03 ^cd^	11.20 ± 0.07 ^e^	20.17 ± 0.04 ^h^	22.05	15.26 (5.68)	17.29 (5.12)	18.19 (4.87)	22.73 (3.90)

All data represent the mean ± standard deviation of triplicate measurements. Different superscript letters within the same column indicate statistically significant differences (*p* < 0.05) among the various treatment and concentration combinations. NTS represents the native tapioca starch, SBL represents a simple blend of TS and hydrocolloid without treatment (control), FT represents freeze–thawing treatment, and CMFT represents critical melting combined with freeze–thawing treatment. All composites were prepared at hydrocolloid concentrations of 0.1%, 0.3%, or 0.5% (*w*/*w*, dry starch basis). The CR%, SR%, RC%, and DCL% indicate crystalline region, subcrystalline region, relative crystallinity of sample, and the degree of crystalline loss, respectively.

**Table 3:** Lamellar and short-ordered structure of tapioca starch (TS) and its composites with carboxymethyl cellulose (CMC) or xanthan gum (XG).

Hydrocolloid Types	Treatments	Hydrocolloid Addition (%)	Lamellar Parameter				Short-Ordered Structure	
			q (nm^−1^)	dBragg (nm)	Imax	Ap	R_1047/1022_	R_1022/995_
-	NTS	-	0.64 ± 0.01 ^c^	9.92 ± 0.16 ^a^	234.22 ± 0.13 ^a^	4.55 ± 0.08 ^a^	0.91 ± 0.01 ^a^	1.06 ± 0.03 ^d^
CMC	SBL	0.1	0.64 ± 0.00 ^bc^	9.85 ± 0.06 ^ab^	227.83 ± 0.12 ^b^	3.84 ± 0.04 ^b^	0.89 ± 0.01 ^ab^	1.10 ± 0.00 ^bcd^
		0.3	0.64 ± 0.00 ^bc^	9.84 ± 0.04 ^abc^	215.66 ± 0.22 ^c^	3.74 ± 0.11 ^b^	0.87 ± 0.01 ^abcd^	1.12 ± 0.02 ^bcd^
		0.5	0.64 ± 0.00 ^bc^	9.84 ± 0.04 ^abc^	210.10 ± 0.01 ^d^	3.72 ± 0.01 ^b^	0.87 ± 0.02 ^abcd^	1.12 ± 0.02 ^bcd^
	FT	0.1	0.64 ± 0.00 ^bc^	9.83 ± 0.02 ^abc^	210.03 ± 1.04 ^d^	3.22 ± 0.11 ^cd^	0.87 ± 0.03 ^abcd^	1.10 ± 0.02 ^cd^
		0.3	0.64 ± 0.00 ^bc^	9.82 ± 0.01 ^abc^	203.57 ± 0.38 ^g^	2.86 ± 0.06 ^de^	0.86 ± 0.00 ^abcd^	1.12 ± 0.03 ^bcd^
		0.5	0.64 ± 0.00 ^bc^	9.81 ± 0.00 ^abc^	195.50 ± 0.56 ^h^	2.85 ± 0.78 ^de^	0.86 ± 0.01 ^abcd^	1.12 ± 0.03 ^bcd^
	CMFT	0.1	0.64 ± 0.00 ^bc^	9.78 ± 0.04 ^bc^	172.66 ± 0.08 ^k^	2.61 ± 0.14 ^efg^	0.85 ± 0.01 ^abcd^	1.15 ± 0.01 ^abc^
		0.3	0.65 ± 0.01 ^ab^	9.76 ± 0.06 ^bc^	164.08 ± 0.32 ^l^	2.48 ± 0.01 ^efg^	0.84 ± 0.03 ^abcd^	1.17 ± 0.02 ^ab^
		0.5	0.65 ± 0.01 ^ab^	9.76 ± 0.06 ^bc^	159.58 ± 0.49 ^n^	2.40 ± 0.02 ^efg^	0.83 ± 0.00 ^bcd^	1.21 ± 0.03 ^a^
XG	SBL	0.1	0.64 ± 0.01 ^bc^	9.84 ± 0.04 ^abc^	207.37 ± 0.09 ^e^	3.76 ± 0.04 ^b^	0.88 ± 0.04 ^abc^	1.08 ± 0.05 ^d^
		0.3	0.64 ± 0.00 ^bc^	9.84 ± 0.04 ^abc^	205.27 ± 0.88 ^f^	3.46 ± 0.06 ^bc^	0.87 ± 0.01 ^abcd^	1.10 ± 0.01 ^bcd^
		0.5	0.64 ± 0.00 ^bc^	9.83 ± 0.03 ^abc^	204.85 ± 0.07 ^f^	3.43 ± 0.02 ^bc^	0.87 ± 0.05 ^abcd^	1.10 ± 0.02 ^cd^
	FT	0.1	0.64 ± 0.00 ^bc^	9.82 ± 0.01 ^abc^	204.60 ± 0.04 ^f^	3.17 ± 0.02 ^cd^	0.87 ± 0.03 ^abcd^	1.10 ± 0.02 ^cd^
		0.3	0.64 ± 0.00 ^bc^	9.82 ± 0.01 ^abc^	194.41 ± 0.03 ^i^	2.76 ± 0.04 ^def^	0.86 ± 0.01 ^abcd^	1.12 ± 0.03 ^bcd^
		0.5	0.64 ± 0.00 ^bc^	9.81 ± 0.00 ^abc^	185.55 ± 0.06 ^j^	2.51 ± 0.30 ^efg^	0.83 ± 0.04 ^bcd^	1.16 ± 0.00 ^abc^
	CMFT	0.1	0.64 ± 0.00 ^ab^	9.77 ± 0.06 ^bc^	164.47 ± 0.36 ^l^	2.47 ± 0.01 ^efg^	0.82 ± 0.06 ^cd^	1.16 ± 0.02 ^abc^
		0.3	0.65 ± 0.01 ^ab^	9.73 ± 0.01 ^bc^	160.72 ± 0.01 ^m^	2.35 ± 0.04 ^fg^	0.82 ± 0.04 ^bcd^	1.17 ± 0.01 ^ab^
		0.5	0.65 ± 0.00 ^a^	9.73 ± 0.01 ^c^	157.56 ± 0.05 ^o^	2.24 ± 0.08 ^g^	0.81 ± 0.01 ^d^	1.19 ± 0.07 ^a^

All data represent the mean ± standard deviation of triplicate measurements. Different superscript letters within the same column indicate statistically significant differences (*p* < 0.05) among the various treatment and concentration combinations. Imax: maximum scattering intensity; Ap: peak area; q: scattering vector; dBragg: lamellar repeat distance. NTS represents the native tapioca starch, SBL represents a simple blend of TS and hydrocolloid without treatment (control), FT represents freeze–thawing treatment, and CMFT represents critical melting combined with freeze–thawing treatment. All composites were prepared at hydrocolloid concentrations of 0.1%, 0.3%, or 0.5% (*w*/*w*, dry starch basis).

**Table 4:** Gel texture properties of tapioca starch (TS) and its composites with carboxymethyl cellulose (CMC) or xanthan gum (XG).

Hydrocolloid Types	Treatments	Hydrocolloid Addition (%)	Hardness (gf)	Springiness	Chewiness (gf)	Gumminess (gf)	Cohesiveness	Resilience
-	NTS	-	45.16 ± 5.69 ^h^	0.78 ± 0.01 ^cd^	33.75 ± 4.89 ^h^	43.30 ± 5.89 ^i^	0.96 ± 0.01 ^a^	0.71 ± 0.02 ^c^
CMC	SBL	0.1	76.24 ± 2.88 ^fg^	0.66 ± 0.00 ^f^	38.22 ± 1.75 ^h^	57.52 ± 2.26 ^h^	0.75 ± 0.00 ^i^	0.58 ± 0.01 ^f^
		0.3	85.22 ± 0.74 ^e^	0.68 ± 0.01 ^f^	45.15 ± 0.36 ^g^	66.05 ± 0.18 ^g^	0.78 ± 0.01 ^h^	0.58 ± 001 ^f^
		0.5	85.75 ± 0.64 ^e^	0.73 ± 0.01 ^e^	50.95 ± 0.97 ^fg^	69.75 ± 0.09 ^fg^	0.81 ± 0.01 ^g^	0.62 ± 0.01 ^e^
	FT	0.1	85.83 ± 1.51 ^e^	0.73 ± 0.02 ^e^	51.24 ± 2.55 ^fg^	70.03 ± 1.95 ^fg^	0.82 ± 0.01 ^g^	0.62 ± 0.01 ^e^
		0.3	89.19 ± 0.73 ^de^	0.73 ± 0.01 ^e^	53.25 ± 1.32 ^ef^	72.89 ± 0.52 ^ef^	0.82 ± 0.01 ^g^	0.62 ± 0.01 ^e^
		0.5	93.74 ± 0.31 ^d^	0.75 ± 0.00 ^de^	57.96 ± 0.49 ^de^	76.99 ± 0.25 ^de^	0.82 ± 0.00 ^g^	0.62 ± 0.00 ^e^
	CMFT	0.1	115.82 ± 3.94 ^c^	0.88 ± 0.00 ^ab^	93.57 ± 2.59 ^c^	106.38 ± 2.91 ^c^	0.92 ± 0.01 ^c^	0.78 ± 0.01 ^a^
		0.3	133.02 ± 5.37 ^b^	0.90 ± 0.01 ^a^	109.82 ± 3.53 ^b^	122.47 ± 3.03 ^b^	0.92 ± 0.01 ^c^	0.79 ± 0.00 ^a^
		0.5	134.63 ± 3.02 ^ab^	0.90 ± 0.02 ^a^	114.54 ± 7.02 ^ab^	127.58 ± 4.78 ^ab^	0.95 ± 0.01 ^ab^	0.81 ± 0.01 ^a^
XG	SBL	0.1	72.05 ± 0.37 ^g^	0.68 ± 0.03 ^f^	37.13 ± 1.87 ^h^	54.24 ± 0.50 ^h^	0.75 ± 0.00 ^i^	0.54 ± 0.02 ^g^
		0.3	78.21 ± 0.17 ^f^	0.73 ± 0.01 ^e^	48.79 ± 1.00 ^fg^	66.62 ± 0.43 ^g^	0.85 ± 0.00 ^f^	0.66 ± 0.01 ^d^
		0.5	78.76 ± 0.95 ^f^	0.74 ± 0.01 ^e^	50.40 ± 0.19 ^fg^	68.31 ± 1.07 ^fg^	0.87 ± 0.00 ^ef^	0.66 ± 0.00 ^d^
	FT	0.1	78.83 ± 0.38 ^f^	0.75 ± 0.03 ^de^	51.80 ± 2.18 ^ef^	69.26 ± 0.07 ^fg^	0.88 ± 0.00 ^de^	0.67 ± 0.00 ^d^
		0.3	86.52 ± 4.72 ^e^	0.81 ± 0.01 ^c^	61.49 ± 3.41 ^d^	76.39 ± 3.27 ^de^	0.88 ± 0.01 ^de^	0.71 ± 0.01 ^c^
		0.5	87.32 ± 1.30 ^e^	0.81 ± 0.00 ^c^	63.29 ± 1.15 ^d^	78.31 ± 1.36 ^d^	0.90 ± 0.00 ^d^	0.74 ± 0.01 ^b^
	CMFT	0.1	111.94 ± 0.51 ^c^	0.86 ± 0.02 ^b^	89.84 ± 3.02 ^c^	104.14 ± 1.38 ^c^	0.93 ± 0.01 ^bc^	0.78 ± 0.02 ^a^
		0.3	135.73 ± 0.47 ^ab^	0.88 ± 0.01 ^ab^	111.64 ± 3.59 ^ab^	126.20 ± 1.93 ^ab^	0.93 ± 0.01 ^bc^	0.78 ± 0.02 ^a^
		0.5	139.58 ± 1.98 ^a^	0.89 ± 0.00 ^ab^	116.62 ± 1.00 ^a^	130.66 ± 0.47 ^a^	0.94 ± 0.01 ^bc^	0.80 ± 0.01 ^a^

All data represent the mean ± standard deviation of triplicate measurements. Different superscript letters within the same column indicate statistically significant differences (*p* < 0.05) among the various treatment and concentration combinations. NTS represents the native tapioca starch, SBL represents a simple blend of TS and hydrocolloid without treatment (control), FT represents freeze–thawing treatment, and CMFT represents critical melting combined with freeze–thawing treatment. All composites were prepared at hydrocolloid concentrations of 0.1%, 0.3%, or 0.5% (*w*/*w*, dry starch basis).

## Data Availability

The original contributions presented in the study are included in the article, further inquiries can be directed to the corresponding author.

## Abbreviations

The following abbreviations are used in this manuscript:

CMFT	Critical melting combined with freeze–thawing treatment
FT	Freeze–thawing treatment
NTS	Native tapioca starch
SBL	A simple blend of TS and hydrocolloid without treatment
